# Epithelial HO-1/STAT3 affords the protection of subanesthetic isoflurane against zymosan-induced lung injury in mice

**DOI:** 10.18632/oncotarget.18605

**Published:** 2017-06-22

**Authors:** Ling Wang, Ya-Li Zhao, Ning-Ning Liu, Xiao-Shan Zhu, Qin-Qin Liu, Hai-Yu Mei, Li-Feng Wang, An-Gang Yang, Chun-Fang Gao, Jun-Tang Li

**Affiliations:** ^1^ Department of Anesthesiology, 150th Central Hospital of PLA, Luoyang, Henan 471031, China; ^2^ Centre of Inflammation and Cancer Research, 150th Central Hospital of PLA, Luoyang, Henan 471031, China; ^3^ Department of Respiration, 150th Central Hospital of PLA, Luoyang, Henan 471031, China; ^4^ State Key Laboratory of Cancer Biology, Department of Biochemistry and Molecular Biology, Fourth Military Medical University, Xi’an, Shaanxi 710032, China; ^5^ State Key Laboratory of Cancer Biology, Department of Immunology, Fourth Military Medical University, Xi’an, Shaanxi 710032, China

**Keywords:** acute lung injury, isoflurane, epithelial cells, heme oxygenase-1, signal transducers and activators of transcription 3

## Abstract

Epithelial dysfunction is a key characteristic of acute lung injury (ALI). Isoflurane (ISO) confers lung protection via anti-inflammatory and anti-apoptotic properties. However, the specific role and potential mechanisms of subanesthetic ISO in lung epithelium protection during zymosan-induced ALI remain unclear. In this study, zymosan increased the expression and activity of beneficial heme oxygenase-1 (HO-1) and signal transducers and activators of transcription 3 (STAT3) in the lung and isolated type II alveolar epithelial cells (AECs-II) from wild-type (WT) mice, which was further enhanced by ISO treatment. ISO reduced the mortality, lung edema, histological changes and pulmonary cell apoptosis, and simultaneously decreased total cells, tumor necrosis factor-α (TNF-α) and interleukin-1β (IL-1β) levels in bronchoalveolar lavage fluid in the zymosan-stimulated WT mice but not in HO-1-deficient mice. Moreover, ISO abated zymosan-augmented lactate dehydrogenase activity, TNF-α and IL-1β production, and apoptosis in WT AECs-II but not in HO-1- or STAT3-silenced cells. Mechanisticly, the epithelial protective effects of ISO on zymosan insult *in vivo* and *in vitro* were mediated by a positive feedback loop comprising STAT3 and HO-1. Pro-survival and anti-apoptosis by ISO was highly reliant on activated STAT3, involving in downstream Akt activation and reduced ratio of pro-apoptotic/anti-apoptotic molecules. Overall, HO-1/STAT3 signaling is in favor of lung epithelial protection of ISO in zymosan-challenged mice, suggesting ISO as a valuable therapeutic agent for ALI.

## INTRODUCTION

Acute lung injury (ALI) and its most severe form, namely, acute respiratory distress syndrome (ARDS), are devastating clinical conditions for critically ill patients. ALI/ARDS is mainly characterized by increased inflammation, hypercoagulation, hypofibrinolysis, and vascular and epithelial permeability [1]. ALI/ARDS occurs in approximately 79 per 100, 000 patients annually in the United States and has a mortality of up to 30%–40% [2, 3]. Despite the great improvement in ALI/ARDS, there are limited therapeutic interventions [4]. Thus, identifying the novel pathological mechanisms of ALI/ARDS and developing specific pharmacological treatments are necessary and urgent.

Various types of cells participate in the pathology of ALI/ARDS, and alveolar epithelial cells (AECs) disruption increased lung epithelial permeability [5]. AECs are categorized into flat type I (AECs-I) executing gas exchange and cuboidal type II (AECs-II) excreting pulmonary surfactants; AECs-II serves as distal progenitors for AECs-I development and in immune responses within the alveoli [6]. Reduced AECs-II survival leads to abnormal repair, and consequently acute and chronic pulmonary diseases. However, the biological functions of AECs-II and the underlying mechanisms in ALI remain unknown.

Heme oxygenase-1 (HO-1) is a rate-limiting enzyme in the conversion of heme into carbon monoxide (CO) and biliverdin, which can be induced by various stimuli [7]. HO-1/CO protects against multiple organ injuries, including lung damage [8, 9]. HO-1 activation in the lung exerts protective effects by inhibiting inflammatory, oxidative, and apoptotic signals [10–12]. Reportedly, the signal transducer and activator of transcription (STAT) family proteins are involved in HO-1 activation during hyperoxia; in particular, STAT3 is essential for the protective effects of HO-1 on oxidant-induced lung endothelial injury [13, 14]. STAT3 can be activated by pro- and anti-inflammatory stimuli and participates in signaling pathways that mediate various pulmonary cellular responses to cytokines and growth factors [15, 16]. Overexpression of STAT3C (a constitutive active form of STAT3) in pulmonary epithelium protects against hyperoxic lung injury [17]. STAT3 deletion in pulmonary AECs-II alters the expression of genes that regulate diverse cellular processes, including cell growth and apoptosis [18]. However, the specific roles and functional links between HO-1 and STAT3 in AECs-II during ALI remain unknown.

Isoflurane (ISO) is a widely used inhaled anesthetic with pharmacological properties, such as anti-inflammation, anti-oxidation, and anti-apoptosis [19, 20]. ISO protects against lung injury induced by lipopolysaccharides (LPS), zymosan and cecal ligation and puncture [19–22]. Prospects for clinical usage of ISO (1.2%–2.5%) have been hampered due to its adverse systemic effects [23]; however, our previous studies showed that subanesthetic ISO (0.7%) mitigates zymosan-induced mortality and lung damage in mice by reducing inflammatory responses and reactive oxygen species generation in neutrophils [24, 25]. Reportedly, ISO post-treatment further enhances zymosan-induced increase in HO-1 expression and activity in mouse lungs and HO-1 elevation and activation improves pulmonary vascular permeability [20, 22]. Nevertheless, the role of HO-1 signaling in the protection of AECs-II by ISO remains unclear.

In this study, we investigated the role of epithelial HO-1/STAT3 in the protective effects of subanesthetic ISO on zymosan-induced ALI mice. ISO further enhances zymosan-elevated HO-1 expression and activity in the lung and AECs-II of wild-type (WT) mice. HO-1 is a key player in the protection of ISO against zymosan-induced lung epithelial injury *in vivo* and *in vitro*. Moreover, ISO induced STAT3 activation and STAT3 depletion abrogated the protective effects of ISO on zymosan-caused pulmonary epithelial cell damage and apoptosis. A positive feedback loop was found, in which STAT3 mediated the beneficial effects of ISO on zymosan-insulted lung epithelial cells partly depending on HO-1, and *vice versa*. The pro-survival and anti-apoptosis by ISO were reliant on STAT3 during zymosan-caused lung epithelial destruction. Collectively, subanesthetic ISO protects against zymosan-induced lung epithelial injury *in vivo* and *in vitro* through a positive feedback loop involving HO-1 and STAT3 signaling.

## RESULTS

### ISO enhances zymosan-induced HO-1 expression and activity in mouse lung and AECs-II

**Figure 1:**
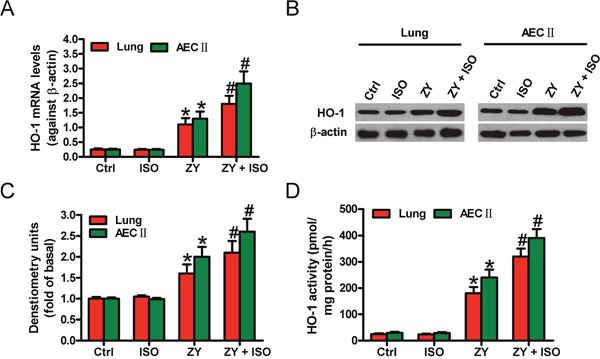
ISO further enhanced zymosan-increased expression and activity of HO-1 in the lung tissues and AECs-II of mice WT mice were i.p. injected with zymosan (1 g/kg) or normal saline, followed by 1 h of 0.7% ISO inhalation at 1 and 6 h after zymosan administration, respectively. All the mice were sacrificed 24 h after zymosan injection. AECs-II were isolated from WT mice and exposed to zymosan (0.5 mg/ml) or culture media for 30 min, followed by 30 min of ISO exposure. Cells were collected at 24 h after zymosan stimulation. **(A, B)** HO-1 mRNA (A) and protein (B) expression levels in lung tissues and AECs-II were detected by qPCR and Western blot assays. β-actin was used as the endogenous control. **(C)** Quantification of HO-1 protein in **(B)**. **(D)** HO-1 activity was determined in lung tissues and AECs-II. Results are represented as the mean ± SD of 3 independent experiments. *P < 0.05 versus Ctrl or ISO group; ^#^*P* < 0.05 versus ZY group. Ctrl: control; ISO: isoflurane; ZY: zymosan.

We firstly found that zymosan upregulates HO-1 mRNA and protein expressions in the lung tissues and AECs-II of WT mice compared with the control group (Figure 1A–1C). Treatment with ISO further increased the mRNA and protein levels of HO-1 (Figure 1A–1C). Moreover, ISO enhanced zymosan-induced increase in HO-1 activity in the lung and AECs-II of WT mice (Figure 1D). These results demonstrated that ISO improves the expression and activity of HO-1 in zymosan-stimulated mouse lung and AECs-II.

### HO-1 plays an essential role in ISO-mediated reduction of zymosan-induced mortality and lung injury *in vivo* and *in vitro*

**Figure 2:**
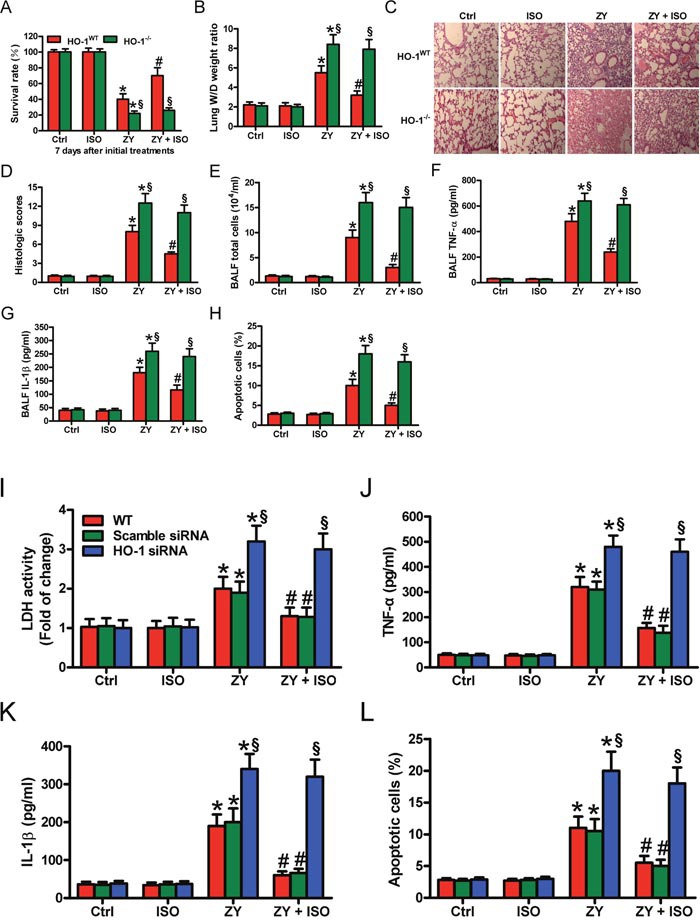
HO-1 is crucial in the protective effects of ISO against zymosan-induced mortality and lung injury *in vivo* and *in vitro* **(A–H)** WT and HO-1^−/−^ mice were i.p. injected with zymosan (1 g/kg) or normal saline, followed by 1 h of 0.7% ISO exposure at 1 and 6 h after zymosan administration, respectively. All the mice were sacrificed 24 h after zymosan insult, except for those in the survival studies. **(A)** The survival rate was evaluated 7 days after zymosan or NS injection. **(B)** Lung W/D ratio was measured. **(C)** Representative HE staining results of lung histopathological changes (100 × magnification). **(D)** Lung histological scores were calculated. **(E)** Total cells in BALF were counted. **(F, G)** The BALF levels of TNF-α **(F)** and IL-1β **(G)** were determined using ELISAs. **(H)** TUNEL staining was performed to detect apoptosis of lung cells and the percentage of TUNEL-positive cells was calculated. **(I–L)** The isolatedWT AECs-II were transfected with scrambled or HO-1 siRNA, then exposed to zymosan (0.5 mg/ml) or culture media for 30 min, followed by 30 min of ISO exposure. Cells were collected 8 or 24 h after zymosan insult. **(I)** LDH activity was measured using a commercially available kit 24 h after zymosan stimulation. **(J, K)** ELISAs were performed to assess the levels of TNF-α **(J)** and IL-1β **(K)** in the supernatants 8 h after zymosan challenge. **(L)** AECs-II apoptosis was detected using flow cytometry 24 h after zymosan treatment. Results are represented as mean ± SD of 3 independent experiments. *P < 0.05 versus Ctrl or ISO group; ^#^*P* < 0.05 versus ZY group; ^§^*P* < 0.05 versus WT or scrambled siRNA group. Ctrl: control; ISO: isoflurane; ZY: zymosan; WT: wild-type.

To investigate the roles of HO-1 in zymosan-caused mouse lung injury model, we first evaluated the survival rate of WT and HO-1-deficiency (HO-1^−/−^) mice. Zymosan-increased mortality is significantly reduced by ISO in WT mice; however, in HO-1^−/−^ mice, the survival rate was not markedly changed in the zymosan + ISO group compared with the zymosan group (Figure 2A). The wet-to-dry (W/D) ratio of WT mouse lung showed a notable decrease in zymosan + ISO group compared with the zymosan group (Figure 2B). HO-1 deficiency eliminated the reductive effect of ISO on lung W/D ratio (Figure 2B). Hematoxylin and eosin (HE) staining results showed that pulmonary pathological changes, characterized by alveolar congestion and inflammatory cell infiltration into the airspace, were markedly inhibited by ISO in zymosan-exposed WT mice but not in HO-1^−/−^ mice (Figure 2C and 2D). Furthermore, the number of total cells in bronchoalveolar lavage fluid (BALF) showed a similar change (Figure 2E). The tumor necrosis factor-α (TNF-α) and interleukin-1β (IL-1β) levels in BALF were also reduced by ISO in zymosan-challenged WT mice but not in HO-1^−/−^ mice (Figure 2F and 2G). The percentage of apoptotic pulmonary cells was significantly decreased by ISO in zymosan-treated WT mice but not in HO-1^−/−^ mice (Figure 2H). Next, we investigated the roles of HO-1 in zymosan-stimulated WT AECs-II using a siRNA approach. The interfering efficiency of HO-1 siRNA and its effect on STAT3 expression see (Supplementary Figure 1). ISO reduced zymosan-increased lactate dehydrogenase (LDH) activity in WT or scrambled siRNA-transfected AECs-II but not in HO-1 siRNA-transfected AECs-II (Figure 2I). ISO suppressed zymosan-enhanced generation of TNF-α and IL-1β in WT or scrambled siRNA-transfected AECs-II, but not in HO-1 siRNA-transfected AECs-II (Figure 2J and 2K). Moreover, ISO attenuated zymosan-induced WT or scrambled siRNA-transfected AECs-II apoptosis, but not HO-1 siRNA-transfected AECs-II (Figure 2L). These data indicated that HO-1 serves an important role in the protective effects of ISO on zymosan-induced lung epithelial injury *in vivo* and *in vitro*.

### STAT3 depletion counteracts the protective effects of ISO against zymosan-induced pulmonary epithelial cell injury and apoptosis

**Figure 3:**
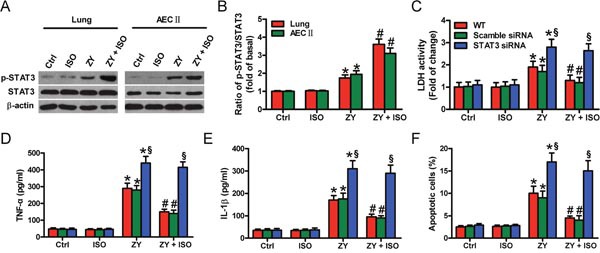
STAT3 activation contributes to ISO-exerted beneficial effects on zymosan-induced lung epithelial cell injury and apoptosis WT mice were i.p. injected with zymosan (1 g/kg) or normal saline, followed by 1 h of 0.7% ISO exposure at 1 and 6 h after zymosan administration, respectively; the mice were sacrificed 24 h after zymosan injection. AECs-II isolated from WT mice were treated with zymosan (0.5 mg/ml) or culture media for 30 min, followed by 30 min of ISO exposure. Cells were collected 24 h after zymosan stimulation. **(A)** Representative Western blot results of p-STAT3 and STAT3 in the lung tissues and AECs-II of WT mice. β-actin was used as the endogenous control. **(B)** The ratios from p-STAT3 to STAT3 **(A)** are shown. **(C–F)** WT AECs-II were transfected with scrambled or STAT3 siRNA, and exposed to zymosan (0.5 mg/ml) or culture media for 30 min, followed by 30 min of ISO treatment. Cells were collected 8 or 24 h after zymosan stimulation. **(C)** LDH activity was measured using a commercially available kit 24 h after zymosan stimulation. **(D, E)** The TNF-α **(C)** and IL-1β **(D)** levels were assessed using ELISAs 8 h after zymosan insult. **(F)** Flow cytometry was performed to evaluate AECs-II apoptosis 24 h after zymosan challenge. Results are represented as mean ± SD of 3 independent experiments. *P < 0.05 versus Ctrl or ISO group; ^#^*P* < 0.05 versus ZY group; ^§^*P* < 0.05 versus WT or scrambled siRNA group. Ctrl: control; ISO: isoflurane; ZY: zymosan; WT: wild-type.

Next, we demonstrated that zymosan activates STAT3 in WT mouse lung and AECs-II, as evidenced by increased phosphorylated (p)-STAT3, which is further enhanced by ISO (Figure 3A and 3B). To determine the functions of STAT3 in ISO-mediated pulmonary epithelial protection, we transfected AECs-II with scrambled or STAT3 siRNA before zymosan challenge. The interfering efficiency of STAT3 siRNA and its effect on HO-1 expression see (Supplementary Figure 2). Consequently, ISO attenuated zymosan-elevated LDH activity in WT or scrambled siRNA-transfected AECs-II but not in STAT3 siRNA-transfected AECs-II (Figure 3C). Also, ISO considerably reduced zymosan-induced release of TNF-α and IL-1β in WT or scrambled siRNA-transfected AECs-II, which was eliminated by STAT3 knockdown (Figure 3D and 3E). ISO hindered zymosan-augmented apoptotic AECs-II, which was neutralized by STAT3 depletion (Figure 3F). These results suggested that ISO protectsagainst zymosan-caused lung epithelial cell damage and apoptosis involving in STAT3 activation.

### STAT3-mediated epithelial protective effects of ISO are dependent on HO-1 *in vivo* and *in vitro*

**Figure 4:**
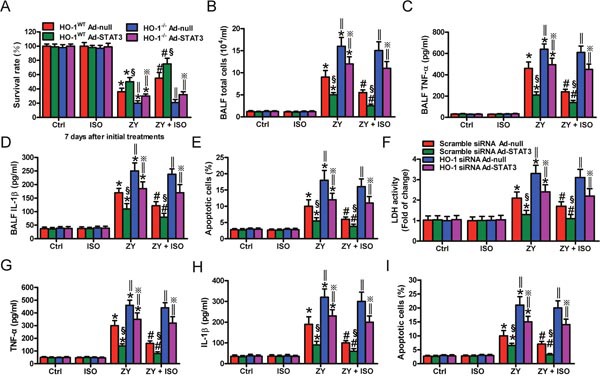
STAT3-mediated lung epithelial protection of ISO during zymosan insult is dependent on HO-1 *in vivo* and *in vitro* **(A–E)** WT and HO-1^−/−^ mice were intranasally administered with Ad-null or Ad-STAT3, and then i.p. injected with zymosan (1 g/kg) or normal saline, followed by 1 h of 0.7% ISO exposure 1 and 6 h after zymosan administration, respectively. All the mice were sacrificed 24 h after zymosan administration, except for those in the survival studies. **(A)** The survival rate was calculated 7 days after zymosan or NS challenge. **(B–D)** The total cell counts **(B)** and TNF-α **(C)** and IL-1β **(D)** levels in BALF were assessed. **(E)** The percentage of cell apoptosis was determined by TUNEL assay. **(F–I)** WT AECs-II were co-treated with scrambled or HO-1 siRNA and Ad-null or Ad-STAT3, and then exposed to zymosan (0.5 mg/ml) or culture media for 30 min, followed by 30 min of ISO treatment. Cells were collected 8 or 24 h after zymosan stimulation. **(F)** LDH activity was assessed 24 h after zymosan challenge. **(G, H)** TNF-α **(G)** and IL-1β **(H)** levels in the supernatants were measured using ELISAs 8 h after zymosan insult. **(I)** Flow cytometry was performed to analyze cell apoptosis 24 h after zymosan administration. Results are represented as mean ± SD of 3 independent experiments. *P < 0.05 versus Ctrl or ISO group; ^#^*P* < 0.05 versus ZY group; ^§^*P* < 0.05 versus HO-1 WT Ad-null group in (A–E) or scrambled siRNA Ad-null group in (F–I); ‖*P* < 0.05 versus HO-1 WT group in (A–E) or scrambled siRNA group in (F–I). Ctrl: control; ISO: isoflurane; ZY: zymosan; WT: wild-type.

STAT3 mitigates hyperoxia-induced lung endothelial injury relying on HO-1 [14]. We investigated whether the STAT3-mediated protective effects of ISO against zymosan-induced lung epithelial damage are dependent on HO-1. STAT3 was overexpressed in WT mouse lung and AECs-II by Ad-STAT3 infection (Supplementary Figure 3). STAT3 overexpression significantly prevented WT mice from zymosan-induced death compared with Ad-null administration, but not improve in that of HO-1^−/−^ mice (Figure 4A). ISO led to a notable increase in the survival rate of zymosan-exposed WT mice; however, ISO had no obvious effect on the survival of zymosan-challenged HO-1^−/−^ mice (Figure 4A). In WT mice, zymosan-elevated total cells and the TNF-α and IL-1β levels in BALF were significantly decreased by STAT3 overexpression, which were further reduced by ISO. In HO-1^−/−^ mice, neither Ad-STAT3 administration nor ISO treatment affected zymosan-enhanced BALF total cells and TNF-α and IL-1β levels (Figure 4B–4D). A similar effect was found in pulmonary apoptotic cells (Figure 4E). Consistent with *in vivo* findings, STAT3 overexpression could not rescue the HO-1 siRNA-transfected WT AECs-II from zymosan-induced injury, as assessed by LDH activity, TNF-α and IL-1β production, and apoptosis (Figure 4F–4I). ISO synergistically enhanced the protective effects of STAT3 in WT AECs-II but not in HO-1-silenced WT AECs-II (Figure 4F–4I). These data indicated that ISO activated epithelial STAT3 depending on HO-1 to exert its protective effects on zymosan-induced lung injury *in vivo* and *in vitro*.

### HO-1-mediated beneficial effects of ISO on zymosan-ruined lung epithelial cells are reliant on STAT3

**Figure 5:**
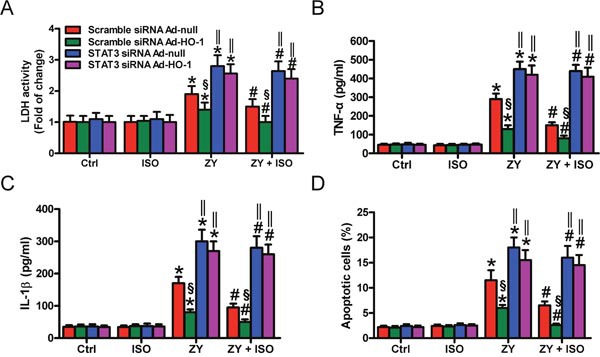
HO-1-mediated pulmonary epithelial protection of ISO during zymosan challenge is reliant on STAT3 AECs-II isolated from WT mice were co-treated with scrambled or STAT3 siRNA and Ad-null or Ad-HO-1, then exposed to zymosan (0.5 mg/ml) or culture media for 30 min, followed by 30 min of ISO treatment. Cells were collected 8 or 24 h after zymosan stimulation. **(A)** LDH activity was assessed 24 h after zymosan treatment. **(B, C)** TNF-α **(B)** and IL-1β **(C)** levels in the supernatants were measured using ELISAs 8 h after zymosan insult. **(D)** Flow cytometry was performed to analyze cell apoptosis 24 h after zymosan challenge. Results are represented as mean ± SD of 3 independent experiments. *P < 0.05 versus Ctrl or ISO group; ^#^*P* < 0.05 versus ZY group; ^§^*P* < 0.05 versus scrambled siRNA Ad-null group; ‖*P* < 0.05 versus scrambled siRNA group. Ctrl: control; ISO: isoflurane; ZY: zymosan; WT: wild-type.

STAT3 contributes to the beneficial effects of HO-1 in oxidant-induced lung endothelial injury [14]. We here investigated whether HO-1-mediated epithelial cell protection of ISO depends on STAT3. HO-1 was overexpressed in WT AECs-II by Ad-HO-1 infection (Supplementary Figure 4). HO-1 overexpression markedly reduced the zymosan-elevated LDH activity in the scrambled siRNA-transfected AECs-II but not in that with STAT3 siRNA transfection, which was further inhibited by ISO treatment (Figure 5A). Compared with the cells infected with Ad-null, Ad-HO-1 infection attenuated zymosan-induced TNF-α and IL-1β production in scrambled siRNA-treated AECs-II but not in STAT3 siRNA-transfected AECs-II (Figure 5B and 5C). ISO further reduced the release of TNF-α and IL-1β in AECs-II co-treated with Ad-HO-1 and scrambled siRNA (Figure 5B and 5C). Zymosan-triggered apoptosis was suppressed by Ad-HO-1 infection and further inhibited by ISO in scrambled siRNA-transfected AECs-II but not in STAT3-silenced AECs-II (Figure 5D). These results revealed that HO-1-mediated protective effects of ISO on zymosan-caused AECs-II injury depend on STAT3.

### The pro-survival and anti-apoptosis effects of ISO on zymosan-challenged lung epithelial cells are reliant on STAT3 *in vivo* and *in vitro*

**Figure 6:**
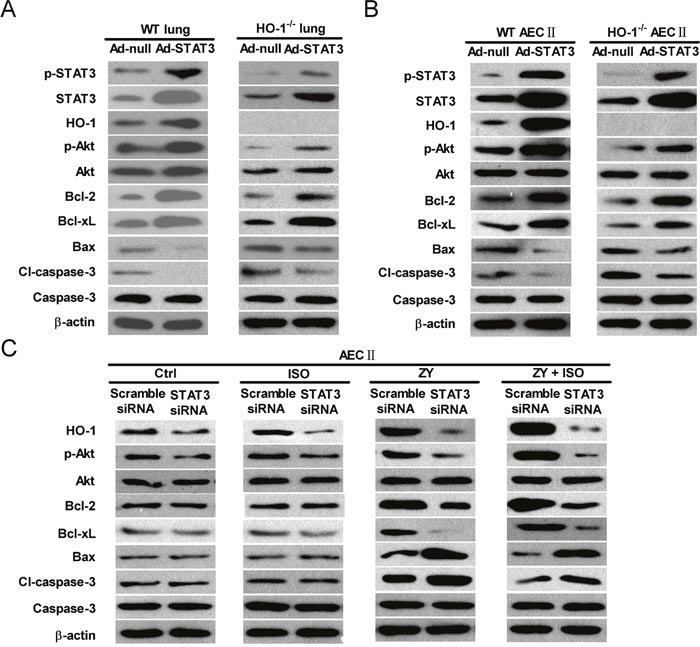
The anti-apoptotic effects of ISO depend on STAT3 in lung epithelial cells *in vivo* and *in vitro* **(A)** WT and HO-1^−/−^ mice were intranasally administered with Ad-null or Ad-STAT3 and sacrificed after 48 h, and lung lysates were immunoblotted against p-STAT3, STAT3, HO-1, p-Akt, Akt, Bcl-2, Bcl-xL, Bax, cl-caspase-3, caspase-3, and β-actin. **(B)** AECs-II isolated from WT and HO-1^−/−^ mice were infected with Ad-null or Ad-STAT3 for 48 h, and cell lysates were immunoblotted against p-STAT3, STAT3, HO-1, p-Akt, Akt, Bcl-2, Bcl-xL, Bax, cl-caspase-3, caspase-3, and β-actin. **(C)** WT AECs-II were transfected with scrambled or STAT3 siRNA, then exposed to zymosan (0.5 mg/ml) or culture media for 30 min, followed by 30 min of ISO treatment. Cells were collected 24 h after zymosan stimulation. Western blot was performed to analyze the expression levels of HO-1, p-Akt, Akt, Bcl-2, Bcl-xL, Bax, cl-caspase-3, caspase-3, and β-actin. β-actin was used as the endogenous control. Data are representative of 3 independent experiments.

To investigate whether the pro-survival and anti-apoptosis effects of ISO are dependent on STAT3, we first examined the expression levels of several apoptosis-related proteins in response to STAT3 overexpression. As shown in Figure 6A and (Supplementary Figure 5A), WT and HO-1^−/−^ mice administered with Ad-STAT3 had increased lung levels of p-STAT3, STAT3, and HO-1, as well as anti-apoptotic proteins p-Akt, Bcl-2, and Bcl-xL. The levels of pro-apoptotic proteins Bax and cleaved (cl)-caspase-3 decreased with STAT3 overexpression. Also, we probed the ability of STAT3 to specifically modulate the apoptosis-associated proteins in AECs-II from WT and HO-1^−/−^ mice. Concomitant with the high expression of STAT3, the protein levels of HO-1, p-Akt, Bcl-2, and Bcl-xL were increased; moreover, Bax and cleaved (cl)-caspase-3 were decreased (Figure 6B and Supplementary Figure 5B). Next, we explored the roles of STAT3 in ISO regulation of these apoptosis-associated proteins in zymosan-stimulated WT AECs-II using a siRNA approach. In WT AECs-II transfected with scrambled siRNA, zymosan led to a significant increase in HO-1, p-Akt, Bcl-2, Bcl-xL, Bax and cl-caspase-3 (Figure 6C and Supplementary Figure 5C). HO-1, p-Akt, Bcl-2, and Bcl-xL protein levels were further enhanced by ISO treatment; however, Bax and cl-caspase-3 protein expression were significantly reduced by ISO treatment (Figure 6C and Supplementary Figure 5C). Zymosan-challenged WT AECs-II with ISO post-treatment showed a decreased ability to upregulate HO-1, p-Akt, Bcl-2, and Bcl-xL, as well as to downregulate Bax and cl-caspase-3 expression in response to STAT3 depletion (Figure 6C and Supplementary Figure 5C). These results indicated that the pro-survival and anti-apoptosis effects of ISO on zymosan-insulted lung epithelial cells depend on STAT3 *in vivo* and *in vitro*.

## DISCUSSION

**Figure 7:**
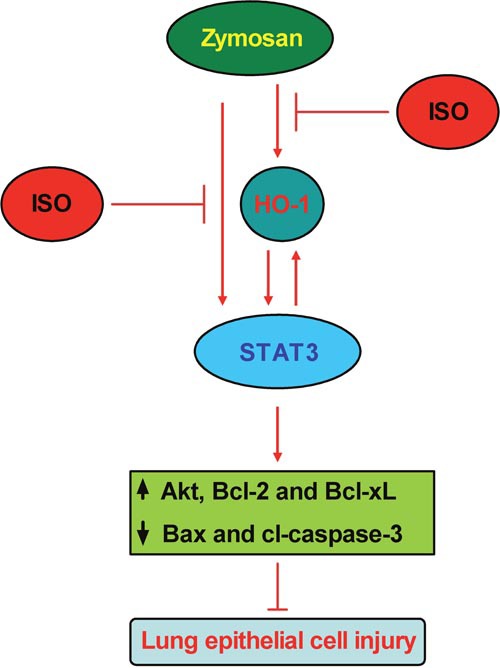
Schematic diagram of the lung epithelial protection of ISO against zymosan-induced ALI involving in HO-1 and STAT3 signaling

Zymosan induces classically pulmonary inflammatory responses, thereby providing a representative model of ALI [20]. Subanesthetic ISO reduces neutrophil inflammatory response in zymosan-induced lung injury [24, 25]. In this study, epithelial HO-1/STAT3 is conducive to the protection of subanesthetic ISO against zymosan-caused lung damage, and several key findings are as follows (Figure 7). First, ISO enhances zymosan-induced HO-1 expression and activity in the lung and AECs-II of WT mice. Second, ISO decreases zymosan-led mortality and lung injury of WT mice but not in HO-1^−/−^ mice, as evidenced by reduction in W/D ratio, total cells and the levels of TNF-α and IL-1β in BALF, and the percentage of apoptotic pulmonary cells. Third, ISO reduces zymosan-increased LDH activity, TNF-α and IL-1β release, and apoptosis of WT AECs-II, which were abrogated by HO-1 knockdown. Fourth, zymosan-activated STAT3 is further enhanced by ISO treatment in the lung and AECs-II of WT mice; moreover, STAT3 silencing attenuated ISO-caused reduction in zymosan-elevated LDH activity, TNF-α and IL-1β release, and apoptosis of WT AECs-II. Fifth, STAT3 overexpression further enhances the beneficial effects of ISO in the lung and AECs-II of zymosan-administrated WT mice but not in that of HO-1^−/−^ mice. Sixth, HO-1 overexpression increases the protective effects of ISO in zymosan-stimulated AECs-II with scrambled siRNA transfection but not in that with STAT3 depletion. Seventh, STAT3 upregulation elevates anti-apoptotic protein expressions and simultaneously decreases the pro-apoptotic protein levels in the lung and AECs-II from both WT and HO-1^−/−^ mice. Lastly, ISO reduces zymosan-increased pro-apoptotic proteins and zymosan-decreased anti-apoptotic proteins depending on STAT3 in WT AECs-II. Together, HO-1/STAT3 signaling is beneficial to the protection of subanesthetic ISO against zymosan-initiated lung injury.

Dysregulated inflammation, inappropriate accumulation of leukocyte and platelets, activation of uncontrolled coagulation pathways, and altered permeability of alveolar barrier remain central pathophysiologic concepts in ALI/ARDS [26]. Although the alveolar barrier includes both endothelial and epithelial cells, the critical role of the epithelium is highlighted by data that changes in epithelial permeability alone are sufficient to cause pulmonary edema [27]. AECs-II, as alveolar epithelial stem cells, can transdifferentiate into AECs-I that restores the integrity of the alveolar-capillary barrier and normal AEC function [28]. AECs-II determine the pathological turnover after hyperoxia-induced lung injury [29]. Therefore, preventing AECs-II damage may provide insights for improving ALI/ARDS outcomes. Reportedly, ISO attenuates zymosan-induced lung injury by inhibiting inflammation and apoptosis and subsequently improving pulmonary epithelial permeability [20]. In this study, we demonstrated that ISO exerts pulmonary protective effects by reducing the lung W/D ratio, histological changes, apoptotic cells, and BALF total cells and TNF-α and IL-1β levels in zymosan-exposed mice *in vivo*, as well as by inhibiting LDH activity, TNF-α and IL-1β release, and the apoptosis of zymosan-stimulated AECs-II *in vitro*. The above results suggested ISO prevents zymosan-induced lung epithelial injury by hampering inflammation and apoptosis *in vivo* and *in vitro*.

HO-1 is a stress-inducible enzyme which protective effects attribute to decreasing the harmful heme and producing the metabolites CO and bilirubin [30]. The induction of HO-1 occurs as an adaptive and beneficial response in many varied tissues and cellular injury models [31]. HO-1 elevation exists in lung tissue from ARDS patients and in many lung injury models [8, 30]. Exogeneous HO-1 administration reduces pulmonary edema, parenchymal inflammation, and apoptosis in hyperoxic rats [32]. Fujita et al. [33] reported that HO-1^−/−^ mice have increased mortality after lung ischemia, which could be reduced by CO treatment. At the cellular level, highly expressed HO-1 in human pulmonary epithelial cells confers hyperoxia resistance [34], whereas lung epithelial cells isolated from HO-1^−/−^ mice exhibit high susceptibility to Corexit 9500A-induced inflammation, permeability, and apoptosis [35]. Previously, we demonstrated that zymosan increases the expression and activity of HO-1 in the lung of mice, which are further enhanced by ISO [20]. Consistently, in this study, we found that zymosan upregulates HO-1 at mRNA and protein levels and increases in HO-1 activity, which were further augmented by ISO treatment. The pulmonary protective effects of ISO were not observed in HO-1^−/−^ mice or in the HO-1-silenced WT AECs-II, suggesting that epithelial HO-1 plays a key role in the beneficial effects of ISO against zymosan-induced lung injury.

STAT3 is widely expressed in different types of cells and plays a dual role in the modulation of cell proliferation, apoptosis, and inflammation [36]. Conditional ablation of STAT3 in mouse respiratory epithelium does not alter lung morphogenesis and function but enhances susceptibility to hyperoxia and adenoviral infection [37, 38]; whereas STAT3C overexpression in pulmonary epithelium protects against hyperoxic lung injury [17], implying that STAT3 is required for maintaining surfactant homeostasis and lung epithelial function. STAT3 activation can inhibit human bronchial epithelial cell apoptosis in response to cigarette smoke exposure [39]. Ao et al. [40] reported that STAT3 activation is involved in the protective effects of vasoactive intestinal peptides against hyperoxia in murine alveolar epithelial MLE-12 cells. In the present study, we demonstrated that STAT3 is activated in the lung and AECs-II of zymosan-treated WT mice, which activation was further increased by ISO treatment. STAT3 knockdown eliminated the protective effects of ISO on zymosan-induced LDH activity, and TNF-α and IL-1β release in WT AECs-II AECs-II, indicating that the beneficial functions of ISO in zymosan-induced lung epithelial injury involved STAT3 activation.

STAT3 regulates cell apoptosis through multiple mechanisms. Many genes modulating cell survival/apoptosis are altered in STAT3-deleted AECs-II, including Akt, caspase-3, and Bcl-2 family members (Bcl2, Bax, Mcl1, and Bad) [18]. Akt is upregulated in cells that constitutively express STAT3 and is downregulated after STAT3 depletion [41]. Intriguingly, Akt is highly involved in the resistance to hyperoxia-induced pulmonary epithelial cell apoptosis [42]. STAT3 is important for mediating the anti-apoptotic effects of CO during anoxia-reoxygenation injuries [43]. Consistent with previous reports, we found that STAT3 overexpression and activation increased anti-apoptotic protein expressions (p-Akt, Bcl-2, and Bcl-xL) and simultaneously decreased pro-apoptotic molecule levels (Bax and cl-caspase-3) in the lung and AECs-II in both WT and HO-1^−/−^ mice. The anti-apoptotic effects of ISO were abrogated by STAT3 silencing in zymosan-exposed WT AECs-II. Overall, the protection of ISO against zymosan-induced lung epithelial cell apoptosis is mediated by STAT3.

The correlation of HO-1 and STAT3 appears to be complex, in which HO-1 functions both upstream and downstream of STAT3 [14]. The functional links between HO-1 and STAT3 are diverse and sometimes contradictory. For example, HO-1 attenuates imiquimod-induced psoriasiform inflammation by negatively regulating STAT3 signaling [44]. HO-1 exhibits anti-inflammatory activity in ovalbumin-induced neutrophilic airway inflammation by inhibiting STAT3 phosphorylation [45]. Inversely, HO-1 induces STAT3 activation by regulating PI3K/Akt signaling, which provides a negative feedback mechanism for TLR4-driven inflammation in mouse liver ischemia/reperfusion injury [46]. Additionally, STAT3 partially mediates HO-1 gene transcription in RAW 264.7 cells during hyperoxia [13]. A positive feedback loop of HO-1 and STAT3 was observed in the lung protective effects during hyperoxia, in which the beneficial effect of HO-1 depends on endothelial STAT3 and *vice versa*. [14]. In this study, we found that HO-1 deficiency eliminated the STAT3-mediated protective effects of ISO in lung epithelial cells *in vivo* and *in vitro*. STAT3 depletion counteracted the HO-1-mediated beneficial effects of ISO in AECs-II. Together, a positive feedback loop of HO-1 and STAT3 contributes to the protection of ISO against zymosan-induced lung epithelium damage.

The current study has several limitations. (1) We should select more time points to investigate the more detailed molecular events *in vitro* and *in vivo*. (2) We here found that the STAT3 effect is slightly HO-1 dependent in terms of the apoptosis endpoints, given that similar findings are seen with or without HO-1 expression. It is speculated that other STAT3 target is important for mediating the above effect. Thus, further studies are needed to clarify the speculation. (3) In this study, we only focused on the mortality rate of mice at 7 days after zymosan or NS injection. Our previous study presented Kaplan-Meier curves so as to be able to analyze the timing of when death of the animals occurred; however, further studies are needed to analyze survival rate of mice in this model. (4) The relations between HO-1 and STAT3, and the effects of these molecules should be further examined well. Because both HO-1 and STAT3 target anti-oxidant genes, we shall examine them in the next studies. (5) Unlike most known anti-inflammation agents that induce inhibition of zymosan-evoked peritonitis, such as morphine and TNF-α-stimulated gene 6 protein, ISO can permeate cell membranes and successfully target organelles, including the cytosol, mitochondria, and nuclei. And now there are no ways of specifically inhibiting ISO. Thus, it is difficult to determine the ISO-specific effect on zymosan-induced HO-1 and STAT3-mediated lung epithelial injury. We speculated that ISO have direct or indirect effects on zymosan-induced lung epithelial cell apoptosis; however, more studies are needed to clarify the speculation. (6) Cell death comprises necroptotic cell death, apoptotic cell death, and pyroptosis. Our previous studies showed that zymosan mainly induced lung cell apoptosis. Therefore, in the present study, we further demonstrated that zymosan induced lung epithelial cell apoptosis and raised the possibility that apoptotic cell death mainly accounted for LDH release from cells. (7) The *in vitro* findings might not completely translate to an *in vivo* situation. Limitations including aging, types of animal, treatment protocol, and the timing periods of administration are critical for evaluating the therapeutic effects of ISO. Thus, more investigations should be performed to grant ISO as a potential therapeutic target of inflammation-caused lung injury.

In summary, the data presented here show that ISO is critical in lung epithelial protection during zymosan-induced ALI. ISO improved mouse survival and reduced lung LDH activity, histological changes, inflammatory responses, and AECs-II apoptosis *in vivo* and *in vivo*. The protective effects of ISO on the lung epithelium was mediated by a positive feedback loop involving in HO-1 and STAT3, in which the STAT3-mediated beneficial effects of ISO were dependent on epithelial HO-1 and *vice versa*. This study uncovered a novel mechanism of zymosan-induced lung epithelial dysfunction and provided the rationale for treating lung injury with ISO.

## MATERIALS AND METHODS

### Animals and ethics statement

Six-week-old male BALB/c mice were obtained from Laboratory Animal Center of Henan Province (Zhengzhou, Henan, China). All animals were housed under specific pathogen-free conditions with a 12 h light/dark cycle at 22°C–24°C. Standard laboratory chow and tap water were obtained *ad libitum*. This study was performed in strict accordance with the Care and Use of Laboratory Animal Guides by the National Institute of Health (NIH Publication No. 85–23, revised 1996). The experimental protocols were approved by the Ethics Committee of the 150th Central Hospital of PLA. All efforts were made to minimize suffering. Enthanasia by sodium pentobarbital was consistent with the American Veterinary Medical Association Guidelines on Enthanasia, June 2007.

### Generation of HO-1^−/−^ mice

HO-1^−/−^ mice were generated by targeted disruption of the HO-1 gene as previously described [47] and the details are in the supplementary materials and methods. Colonies of mice were maintained by breeding HO-1^−/−^ males with HO-1^+/−^ females. Offspring were genotyped at the time of weaning using PCR to amplify the WT and mutant alleles of genomic DNA from tail samples. WT mice were used as controls.

### Zymosan-induced lung injury and ISO treatment

A zymosan-induced lung injury model was established by asepticly intraperitoneally (i.p.) injection of zymosan into mice at a dose of 1 g/kg of body weight, as previously described [20]. The same volume of normal saline (NS) was injected through the same route to serve as the sham control. To verify the functional role of 0.7% ISO, the WT and HO-1^−/−^ mice were placed in a sealed Plexiglass chamber with inflow and outflow outlets and ISO was delivered by air flow into the chamber through a tube at a rate of 4 L/min. The flow rate of ISO was accurately controlled in real-time by regulation of Anesthetic Vaporizers (Harvard apparatus, USA). ISO concentration in the outflow hose of the chamber was continuously monitored with a gas analyzer (Brϋel & Kjaer, Naerum, Denmark) and maintained at 0.7% during the treatment. Oxygen concentration in the chamber was maintained at 21% using supplemental oxygen and continuously monitored with a gas analyzer (Medical Gas Analyzer LB-2, Model 40 M; Beckman, Fullerton, CA, USA). Carbon dioxide was removed from the chamber gases with Baralyme (Allied Healthcare Products, Inc., St. Louis, MO, USA). Animals without ISO treatment were exposed to room air (RA) in the chamber as the vehicle control. The room and chamber temperatures were maintained within 22°C–24°C.

### *In vivo* experimental design

Eighty WT mice and another Eighty HO-1^−/−^ mice were randomly allocated as follows, respectively (each group = 20): (1) Zymosangroup: WT and HO-1^−/−^ mice were given an i.p. injection of zymosan, followed by inhalation of RA for 1 h starting at 1 h and 6 h after zymosan administration. (2) Zymosan + ISO group: no differences from the zymosan group, except for 1 h inhalation of 0.7% ISO starting at 1 h and 6 h instead of RA after zymosan administration. (3) Control (Ctrl) group: no differences from the zymosan group, except for administration with NS instead of zymosan. (4) ISO group: identical to the Ctrl group, except for 1 h inhalation of 0.7% ISO starting at 1 h and 6 h instead of RA after NS administration. All the mice were euthanized at 24 h after zymosan or NS administration, except for those in the survival studies. The survival rate was evaluated 7 days after zymosan or NS injection.

### Mouse AECs-II isolation, culture, and treatment

AECs-II were isolated from WT and HO-1^−/−^ mice using modified methods by Gereke et al. [48] and the details are in the supplementary materials and methods. For ISO treatment, AECs-II were seeded on 6-well plates, allowed to incubate overnight, and subjected to zymosan (0.5mg/ml) or control culture media treatment for 30 min, and the media volume in each well was reduced from 2.5 ml to 1 ml and the cells were exposed to RA with or without ISO at 2 L/min in a metabolic chamber (Columbus Instruments) for 30 min. During ISO exposure, the ISO concentration (0.7%) was continuously verified by sampling exhaust gas using a DatexCapnomac (SOMA Technology Inc., Cheshire, CT, UK). After AECs-II were treated with zymosan or culture media for the indicated times, the below assays were carried out.

### Transit transfection with siRNAs

AECs-II were seeded into 6- or 12-well plates. After reaching 50%–60% confluence, the cells were transfected with 100 nM of HO-1 (sc-35555) or STAT3 (sc-423176) siRNAs (all from Santa Cruz Biotechnology Inc., Dallas, TX, USA) using Lipofectamine^®^ 2000 (Invitrogen, Carlsbad, CA, USA) according to the manufacturer's instructions.

### Overexpression of HO-1 and STAT3

The recombinant adenoviral constructs encoding mouse HO-1 (Ad-HO-1) and STAT3 (Ad-STAT3) and empty vector (Ad-null) were purchased from Vector Biolabs (Malvern, PA, UK). Adenovirus was plaque-purified and propagated in packaging human embryonic kidney 293A cells, and purified and titrated using Adeno-X Virus Kits (Clontech, Palo Alto, CA, USA) in accordance with the manufacturer's protocols. The virus was stored at –80°C until use. Ad-null was used as the control. For *in vivo* experiments, mice intranasally received Ad-STAT3 or Ad-null at an amount of 1 × 10^9^ plaque-forming units suspended in 100 μl of phosphate-buffered saline (PBS). For *in vitro* studies, the AECs-II was stably infected with Ad-HO-1, Ad-STAT3, or Ad-null at 2.5 multiplicity of infection.

### Lung W/D weight ratio

The lung W/D weight ratio was measured to evaluate pulmonary edema. Fresh lungs were harvested and weighed, then placed in an oven at 80°C for 24 h and weighed again when dried. The lung W/D ratio was calculated as the following formula: W/D ratio = (wet weight – dry weight)/dry weight.

### Bronchoalveolar lavage (BAL)

BAL was performed twice with 1 ml PBS (pH 7.4). BALF was centrifuged at 1000 *g* and 4°C for 8 min. Cell pellets were pooled and resuspended in PBS, and the total cells was counted using a hemocytometer (Beckman Coulter, Inc.). The supernatant was collected for TNF-α and IL-1β measurement.

### Histopathological analysis

Lungs were harvested for observing morphologic alterations at 24 h after zymosan or NS administration. The subjects were fixed with 10% formalin for 8 h at room temperature, embedded in paraffin, and sectioned at 4 μm thickness. After deparaffinization and rehydration, the sections were sequentially stained with hematoxylin and eosin. Histologic changes were evaluated by two independent pathologists, who had no knowledge of the treatment regimen received by each respective animal. The degree of lung injury was scored on a subjective scale ranging from 0 to 3; 0 = absence, 1 = mild, 2 = moderate, and 3 = severe. The ranging scale was used for each of histologic features: edema, hyperemia and congestion, neutrophil margination and tissue infiltration, intra-alveolar hemorrhage and debris, and cellular hyperplasia. The final score will be the adding of the single evaluation [24].

### Terminal deoxynucleotidyl transferase dUTP nick end-labeling (TUNEL) assay

TUNEL assay was performed to detect cell apoptosis *in situ*, according to the manufacturer's instruction (Roche, Mannheim, BW, Germany). In brief, lung tissue sections (4 μm) were deparaffinized in xylene, rehydrated in a graded series of ethanol solutions, rinsed with PBS, and incubated with fluorescein isothiocyanate (FITC)-labeled terminal deoxynucleotidyl transferase nucleotide mix at 37°C for 60 min. Subsequently, the sections were rinsed twice with PBS and counterstained with 10 mg/ml 4′, 6-diamidino-2-phenylindole (DAPI; Sigma). TUNEL-positive cells were imaged and mounted using a fluorescent microscope (Carl-Zeiss) and ultimately expressed as a percentage of the total cells determined by DAPI staining.

### Quantitative real-time polymerase chain reaction (qPCR) assay

Total RNA from lung tissues was extracted using the TRIzol reagent (Invitrogen) and reverse transcribed into first-strand cDNA using a SuperScript Reverse Transcriptase kit (Invitrogen) according to the manufacturer's instructions. qPCR assay was performed using an Applied Biosystems 7500 Fast Real-Time PCR System (Applied Biosystems, Foster City, CA, USA). The primers are listed as follows: for HO-1, 5'-CGAATGAACACTCTGGAGATGACAC-3' (forward), and 5'-CCTCTGA CGAAGTGACGCCATC TGT-3' (reverse); for β-actin, 5'-GTGGGCCGCTCTAGG CACCAA-3' (forward), and 5'-CTCTTTGATGTCACGCA CGATTTC-3' (reverse). Gene expression was quantified using 2^-ΔΔCt^ method and normalized with the internal control β-actin.

### Enzyme-linked immunosorbent assay (ELISA)

TNF-α and IL-1β levels in the BALF and AECs-II supernatants were measured using commercially available ELISA kits (Minneapolis, MN, USA), according to the manufacturer's instructions. The optical density (OD) was measured at 490 nm on an ELISA plate scanner (Molecular Devices, Sunnyvale, CA, USA).

### HO-1 activity assay

HO-1 activity was quantified by the spectrophotometric determination of bilirubin generation as previously described [20]. Briefly, the supernatant from homogenated lung tissue and AECs-II was incubated with nicotinamide adenine dinucleotide phosphate (NADPH; 20 mM), hemin (10 mM), and 1 mg of liver cytosol protein (source of bilirubin reductase) for 1 h in a 37°C water bath in the dark. After chloroform extraction, bilirubin formation was determined from the absorbance difference at 464 and 530 nm. Values were expressed as pmol of bilirubin/mg protein/h.

### Measurement of LDH activity

The LDH activity in the AECs-II with different treatments was detected using a commercially available kit (Nanjing Jiancheng Bioengineering Institute, Nanjing, Jiangsu, China) according to the manufacturer's instructions, and expressed as U/L, and ultimately calculated as fold changes compared to Ctrl or ISO group.

### Apoptosis assay by flow cytometry

The apoptosis assay was performed using Annexin V-FITC (BD Bioscience) and propidium iodide (PI; Beyotime, Haimen, China) staining. After the different treatments, cells were harvested, centrifuged, and resuspended in binding buffer. Approximately 10 μl of ready-to-use Annexin V-FITC was added in the mixture, incubated at 37°C for 15 min, and counterstained with 5 μl of PI in the dark for 30 min. Annexin V-FITC and PI fluorescence were assessed by BD FACSCalibur flow cytometry (BD Bioscience). Results were analyzed by CellQuest software (BD Bioscience).

### Western blot analysis

Protein was extracted from lung tissue or AECs-II using RIPA lysis buffer (Beyotime), separated by sodium dodecyl sulfate polyacrylamide gel electrophoresis, electrotransferred to nitrocellulose membranes (Millipore, Boston, MA, USA), and then immunoblotted with primary antibodies against HO-1, STAT3, p-STAT3 (Tyr705), p-Akt (Ser473), Akt (all from Cell Signaling Technology Inc., Danvers, MA, USA), Bcl-2, Bcl-xL, Bax, caspase-3, cl-caspase-3 (all from Santa Cruz), and β-actin (Sigma); followed by incubation with appropriate horseradish peroxidase-conjugated secondary antibodies (Sigma). Detection was performed with an enhanced chemiluminescence assay kit (Pierce, Rockford, IL, USA). All experiments were performed in triplicate.

### Statistical analysis

Data are expressed as the mean ± standard deviation (SD). For parametric data, comparison of different groups was performed by one-way analysis of variance, followed by Tukey's post hoc test for multiple comparisons. Survival rate was plotted using Kaplan−Meier method and analyzed using Log-rank test. Statistical analysis was performed using GraphPad Prism 5 (GraphPad Software Inc., San Diego, CA, USA). *P* < 0.05 was considered statistically significant.

## SUPPLEMENTARY MATERIALS FIGURES



## Abbreviations

ALI: acute lung injury
AECs: alveolar epithelial cells
AECs-I: type I AECs
AECs-II: type II AECs
ARDS: acute respiratory distress syndrome
BALF: bronchoalveolar lavage fluid
CO: carbon monoxide
ELISA: enzyme-linked immunosorbent assay
FITC: fluorescein isothiocyanate
HE: hematoxylin and eosin
HO-1: heme oxygenase-1
HO-1^−/−^: HO-1-deficiency
IL-1β: interleukin-1β
ISO: isoflurane
LDH: lactate dehydrogenase
LPS: lipopolysaccharide
NS: normal saline
PBS: phosphate-buffered saline
PI: propidium iodide
qPCR: quantitative real-time polymerase chain reaction
STAT3: signal transducers and activators of transcription 3
TNF-α: tumor necrosis factor-α
TUNEL: terminal deoxynucleotidyltransferase dUTP nick end-labeling
W/D: wet-to-dry
WT: wild-type.
