# Supplementary material for: Epithelial HO-1/STAT3 affords the protection of subanesthetic isoflurane against zymosan-induced lung injury in mice
Source: Oncotarget. 2017 Jun 22;8(33):54889–903. doi: 10.18632/oncotarget.18605 (PMC5589628; doi:10.18632/oncotarget.18605)
Supplement: Supplementary file 1 [file oncotarget-08-54889-s001.pdf]

Epithelial HO-1/STAT3 affords the protection of subanesthetic isoflurane against zymosan-induced lung injury in mice

SUPPLEMENTARY MATERIALS

Targeted disruption of HO-1 in embryonic stem cells and generation of HO-1$^{-/-}$ mice

To make the HO-1 targeting construct, genomic clones were isolated from a 129 SvJ mouse λDASH II genomic library (Stratagene, La Jolla, California, USA). Subcloning was performed according to the published HO-1 restriction map [1]. The plasmid pHO-neo was constructed by inserting the *Xho*I-*Bam*HI neomycin-resistant gene (*neo*) expression cassette from pMC1neo polyA (Stratagene) into pBluescript II SK (Stratagene), followed by an *Xho*I HO-1 genomic fragment containing 3 kb of 5′ upstream sequence at the *Xho*I site. A *Bam*HI-*Eco*RI HO-1 fragment containing 3 kb of 3′ downstream sequence was inserted into thymidine kinase gene (*TK*) plasmid pPGK-TK. A 6-kb *Cla*I (filled in)-*Bam*HI fragment containing a TK expression cassette and the 3′ arm of the HO-1 genomic sequence was subsequently subcloned into the *Xba*I (filled in)-*Bam*HI site of pHO-neo to generate the HO-1 targeting construct. Then, the construct was linearized with *Not*I before electroporation into D3 embryonic stem (ES) cells. Clones were selected in growth medium containing G418 and ganciclovir [2]; moreover, correctly targeted ES clones were identified by Southern blot analysis. These targeted ES cells were injected into BALB/c and C57BL/6 blastocysts to generate chimeric mice. Male chimeras were mated to BALB/c females, and heterozygous offspring were intercrossed to generate HO-1$^{-/-}$ mice. Mice used for the experiments were maintained on a 129 Sv × BALB/c mixed genetic background.

Isolation and culture of mouse AECs-II

For AECs-II isolation, mice were anaesthetized and exsanguinated by cutting the left and right jugular veins, as well as the renal artery. Lungs were cleared of blood by perfusion with 10 ml of PBS via the pulmonary artery. The trachea was cannulated with the sheath of 22G intravenous catheter. Through the cannula, 2 ml of Dispase II (reconstituted in PBS at 50 U/ml) was infused into the lungs, followed by instillation of 0.5 ml liquefied agarose. The lungs were covered with laboratory tissue paper, placed on ice for 2 min, dissected out from the thoracic cavity, and rinsed with PBS. The lung was dissected and incubated with 2 ml Dispase II at room temperature for 45 min for digestion. After removing the Dispase II, the lung was transferred into a dish with 7 ml DMEM containing anti-CD16/32 antibody (1 µg/ml final concentration) and 100 µl DNase. The lung tissue was completely disintegrated using forceps and incubated for 10 min at room temperature with gentle rocking on a rocker set to 200 rpm. The cell suspensions were filtered sequentially through 70, 48, and 20 µm strainers, spun down, and resuspended in complete DMEM. The filtrate was centrifuged at 160 *g* and 4 °C for 15 min and the supernatant was removed. The cell pellet was resuspended in 2 ml erythrocyte lysis buffer (0.15 M NH_4Cl, 0.01 M $KHCO_3$, 0.1 mM EDTA, pH 7.2) and then lysis was quickly terminated by adding 13 ml DMEM, followed by centrifugation at 160 *g* and 4 °C for 12 min. AECs-II were enriched in this crude lung prep by magnetically depleting cells positive for lineage markers CD45, CD16/32, CD31, Ter119, and integrin β4, which mark hematopoietic, endothelial, erythroid, distal lung progenitor, and club cells [3], respectively. For magnetic depletion, crude lung prep cells were incubated with the anti-lineage and biotinylated antibody cocktail in DMEM for 45 min. Cells were washed to remove excess antibodies and incubated with magnetic Dynabeads (3 µl bead suspension for 1 × 10^6 cells in complete DMEM) on a rotator for 30 min at 4 °C. The lineage-positive cells were removed by placing the cells in the magnetic field of a DynaMag-2 magnet, thereby yielding an AECs-II-enriched suspension. The AECs-II-enriched prep was then retained in complete DMEM for lineage markers using the anti-lineage, biotinylated antibody cocktail, followed by staining with streptavidin-PE and EpCAM-APC as a pan-epithelial surface marker. Cells were washed and suspended in a sorting buffer (PBS with 2% heat-inactivated FBS, 1 mM EDTA, and 1 µg/ml DAPI). Thereafter, DAPI-negative, live, singlet, and EpCAM^{+}-lineage cells were sorted into 15 ml tubes containing complete DMEM. In this sorting strategy, other types of lung cell, likely fibroblasts and smooth muscles, were excluded by being dual-negative for lineage markers and EpCAM. Cell sorting was performed on a BD FACSAria II (BD Biosciences). AECs-II were determined by immunocytochemical labeling with surfactant protein C (Santa Cruz, CA, USA). The purity of the AECs-II used in the study was > 98%. Prior to all experiments, More than 99% of the cells were determined viable using Live/Dead violet (Invitrogen, Carlsbad, CA, USA).

REFERENCES

1. Alam J, Cai J, Smith A. Isolation and characterization of the mouse heme oxygenase-1 gene. Distal 5' sequences are required for induction by heme or heavy metals. The Journal of biological chemistry. 1994; 269:1001-1009.
2. Healy AM, Rayburn HB, Rosenberg RD, Weiler H. Absence of the blood-clotting regulator thrombomodulin causes embryonic lethality in mice before development of a functional cardiovascular system. Proceedings of the National Academy of Sciences of the United States of America. 1995; 92:850-854.
3. Patriarca C, Macchi RM, Marschner AK, Mellstedt H. Epithelial cell adhesion molecule expression (CD326) in cancer: a short review. Cancer treatment reviews. 2012; 38:68-75.

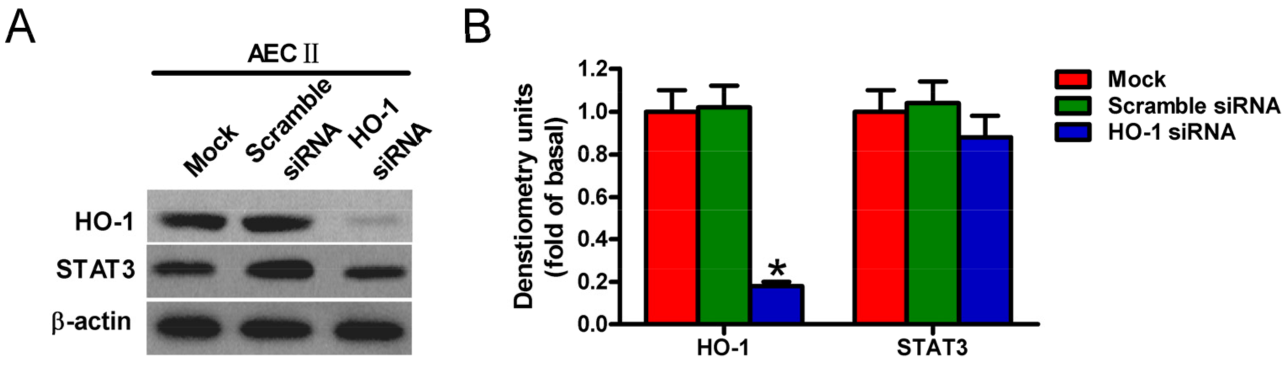

Supplementary Figure 1: The knockdown of HO-1 and its effect on STAT3 expression in AECs-II. AECs-II was isolated from WT mice and transfected with 100 nM of scrambled or HO-1 siRNA for 24 h. **(A)** Western blot analysis was performed to analyze protein expressions of HO-1 and STAT3. β-actin was used as endogenous control. **(B)** Quantification of HO-1 and STAT3 proteins in **(A)**. Results are represented as mean ± SD of 3 independent experiments. *$P < 0.05$ versus mock or scrambled siRNA group.

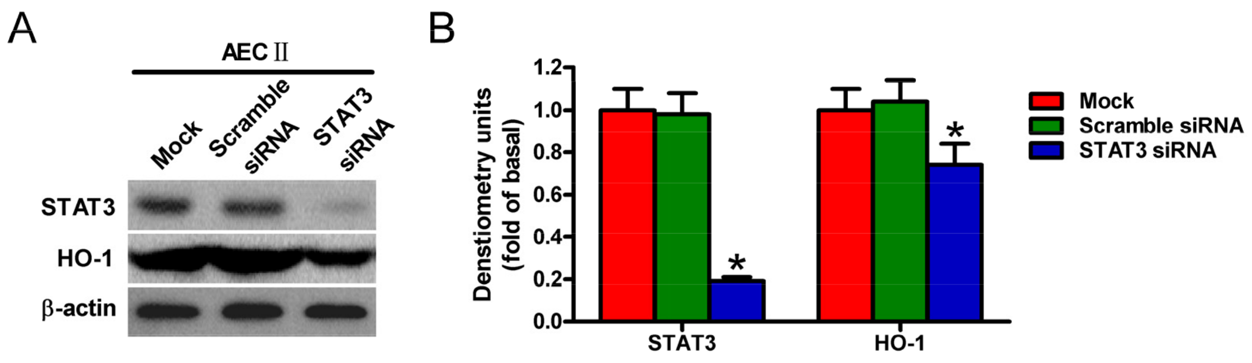

Supplementary Figure 2: The knockdown of STAT3 and its effect on HO-1 expression in AECs-II. AECs-II isolated from WT mice was transfected with 100 nM of scrambled or STAT3 siRNA for 24 h. **(A)** Western blot analysis was performed to analyze protein expressions of STAT3 and HO-1. β-actin was used as endogenous control. **(B)** Quantification of STAT3 and HO-1 proteins in **(A)**. Results are represented as mean ± SD of 3 independent experiments. **P* < 0.05 versus mock or scrambled siRNA group.

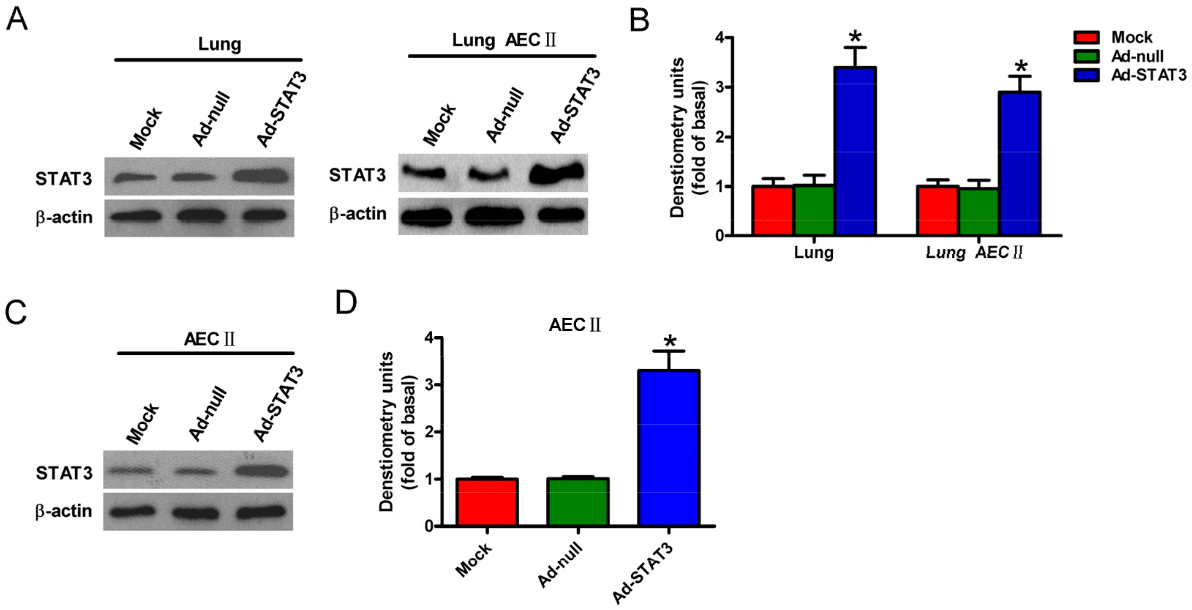

Supplementary Figure 3: The overexpression of STAT3 in mouse lung and AECs-II. **(A)** WT mice were intranasally administered Ad-null or Ad-STAT3 at an amount of 1×10^9 plaque-forming units for 48 h. Representative Western blot results of STAT3 in the lung tissues and isolated lung AECs-II. β-actin was used as endogenous control. **(B)** Quantification of STAT3 protein in **(A)**. **(C)** AECs-II isolated from WT mice was infected with Ad-null or Ad-STAT3 at 2.5 multiplicity of infection for 48 h. Western blot analysis of STAT3 expression. β-actin was used as endogenous control. **(D)** Quantification of STAT3 protein in **(C)**. Results are represented as mean ± SD of 3 independent experiments. **P* < 0.05 versus mock or Ad-null group.

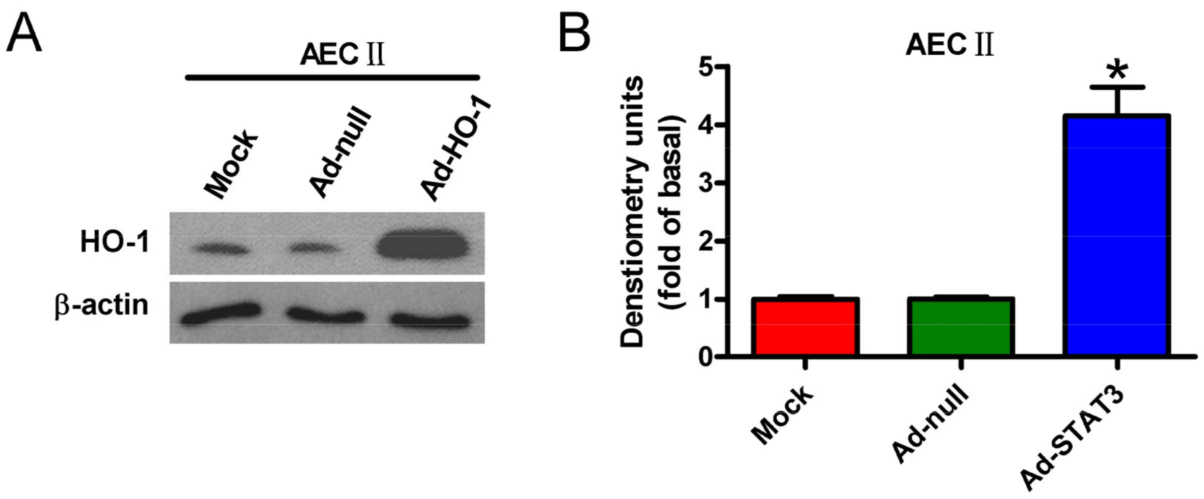

Supplementary Figure 4: The HO-1 overexpression in mouse AECs-II. AECs-II isolated from WT mice was infected with Ad-null or Ad-HO-1 at 2.5 multiplicity of infection for 48 h. Western blot analysis of HO-1 expression. β-actin was used as endogenous control. **(B)** Quantification of STAT3 protein in **(A)**. Results are represented as mean ± SD of 3 independent experiments. **P* < 0.05 versus mock or Ad-null group.

A

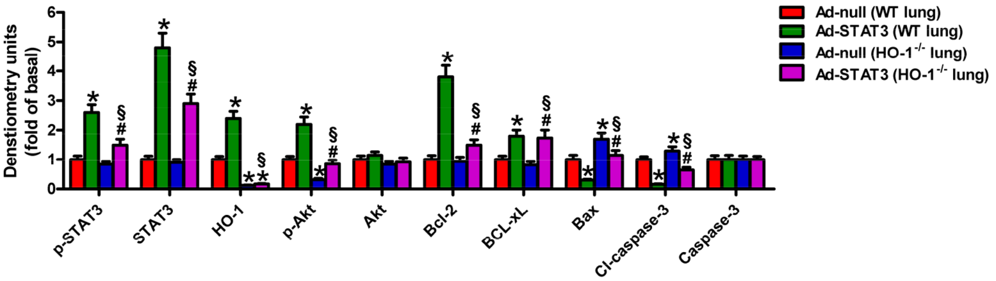

B

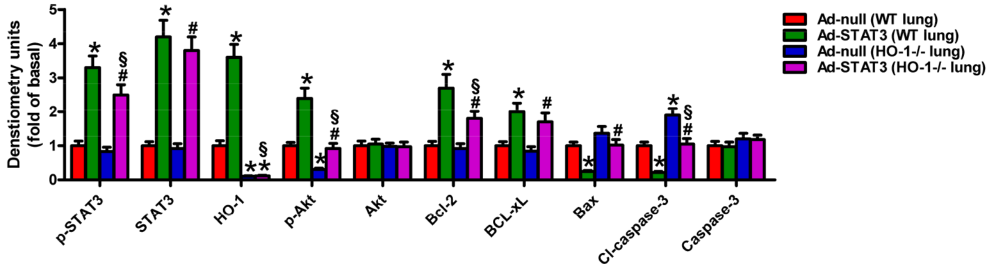

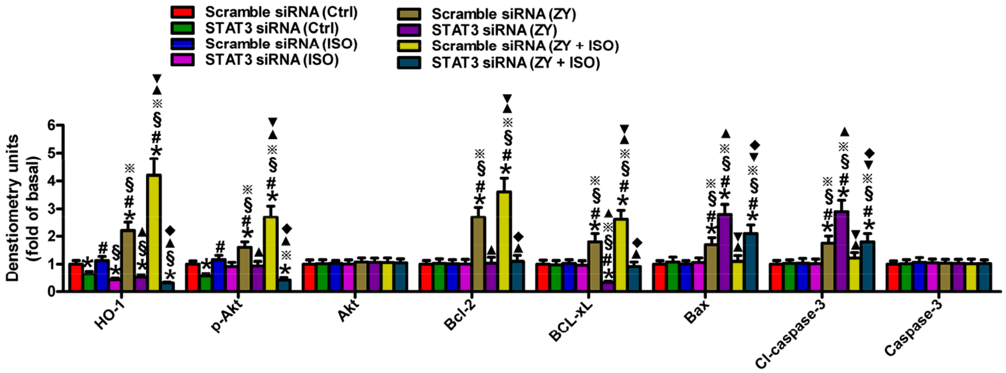

Supplementary Figure 5: Quantification of protein expressions in Figure 6. (A) WT and HO-1$^{-/-}$ mice were intranasally administered with Ad-null or Ad-STAT3 and sacrificed after 48 h, and lung lysates were immunoblotted against p-STAT3, STAT3, HO-1, p-Akt, Akt, Bcl-2, Bcl-xL, Bax, cl-caspase-3, caspase-3, and β-actin. β-actin was used as endogenous control. Quantification of the above proteins is shown. **(B)** AECs-II isolated from WT and HO-1$^{-/-}$ mice were infected with Ad-null or Ad-STAT3 for 48 h, and cell lysates were immunoblotted against p-STAT3, STAT3, HO-1, p-Akt, Akt, Bcl-2, Bcl-xL, Bax, cl-caspase-3, caspase-3, and β-actin. β-actin was used as endogenous control. Quantification of the above proteins is shown. **(C)** WT AECs-II were transfected with scrambled or STAT3 siRNA, then exposed to zymosan (0.5 mg/ml) or culture media for 30 min, followed by 30 min of ISO treatment. Cells were collected 24 h after zymosan stimulation. Western blot was performed to analyze the expression levels of HO-1, p-Akt, Akt, Bcl-2, Bcl-xL, Bax, cl-caspase-3, caspase-3, and β-actin. β-actin was used as the endogenous control. Quantification of the above proteins is shown. Results are represented as mean ± SD of 3 independent experiments. $^{*}P < 0.05$ versus Scramble siRNA (Ctrl) group; $^{\#}P < 0.05$ versus STAT3 siRNA (Ctrl) group; $^{\S}P < 0.05$ versus Scramble siRNA (ISO) group; ※$P < 0.05$ versus STAT3 siRNA (ISO) group. ▲$P < 0.05$ versus Scramble siRNA (ZY) group; ▼$P < 0.05$ versus STAT3 siRNA (ZY) group; ♦$P < 0.05$ versus Scramble siRNA (ZY + ISO) group. Ctrl: control; ISO: isoflurane; ZY: zymosan.
